# Stigmatizing Attitudes Toward Patients With Psychiatric Disorders Among Medical Students and Professionals

**DOI:** 10.3389/fpsyt.2020.00326

**Published:** 2020-04-30

**Authors:** Ana Margarida Oliveira, Daniel Machado, João B. Fonseca, Filipa Palha, Pedro Silva Moreira, Nuno Sousa, João J. Cerqueira, Pedro Morgado

**Affiliations:** ^1^Department of Cardiology, Hospital Senhora da Oliveira, Guimarães, Portugal; ^2^School of Health Sciences, Life and Health Sciences Research Institute (ICVS), University of Minho, Braga, Portugal; ^3^ICVS-3Bs PT Government Associate Laboratory, Braga/Guimarães, Portugal; ^4^Department of Psychiatry, Hospital Senhora da Oliveira, Guimarães, Portugal; ^5^Department of Psychiatry, Hospital de Braga, Braga, Portugal; ^6^ENCONTRAR+SE, Association for the Promotion of Mental Health, Porto, Portugal; ^7^Centre for Studies in Human Development, Faculty of Education and Psychology, Catholic University of Portugal, Porto, Portugal; ^8^Department of Neurology, Hospital de Braga, Braga, Portugal

**Keywords:** stigma, mental health, psychiatrists, students, schizophrenia, AQ-27, mental illness

## Abstract

**Introduction:**

Stigma attached to mental health encompasses discrimination and exclusion of psychiatric patients and hinders their opportunities to have more productive and fulfilling lives. Moreover, stigma also exists among health professionals, and therefore, it hampers the provision of treatment and care and the promotion of mental well-being. This manuscript intends to assess and compare the levels of stigmatization toward patients with mental illness between medical students and doctors from different specialties.

**Methods:**

The Portuguese version of Attribution Questionnaire (AQ-27) was used to assess the attitudes of medical students (n = 203), non-psychiatry doctors (n = 121), and psychiatry specialists (n = 29) from the University of Minho and three hospitals in the region of Braga, Portugal (Hospital de Braga, Hospital Senhora da Oliveira, and Hospital de Fafe).

**Results:**

Psychiatrists were the group that displayed lower levels of stigmatizing attitudes in all the items of the AQ-27, followed by the students. The regression analyses revealed that professional group and presence of a relative with mental illness were the factors that have a significant impact on the levels of stigmatization.

**Conclusions:**

Mental illness stigma is widely spread in community and reaches not only general population but also health professionals. Psychiatrists presented lower levels of stigma compared with non-psychiatry physicians and medical students. We found that stigma is related with age and the presence of relatives with psychiatric disorders. These findings highlight the critical relevance of raising awareness on this topic and, therefore, break stereotypes to reduce the negative consequences of stigma.

## Introduction

Stigma can be defined as a sign that distinguishes a person in a negative way resulting in an additional difficulty for him/her. Stigma toward people with mental health problems consists in an attitude of social disapproval based on certain personal characteristics, beliefs, or behaviors that are in conflict to the sociocultural norm (1). These may be viewed as marks of disgrace and discredit leading a person to be casted away from a standard group (2).

The process of stigmatization may be understood under the social attributions model that establishes a causal relationship between stigma signals, stereotypes, and discriminatory behavioral responses (3). In accordance to this paradigm, the discriminative cues are given by persons suffering from psychiatric disorders through their symptoms, skill deficits, and appearance. Then, the general public will generate impressions and expectations regarding these individuals that are commonly seen as dangerous or responsible for causing their illness (controllability and responsibility). Therefore, these negative beliefs give rise to a wide range of stigmatizing discriminatory attitudes including coercion (mandatory treatment), segregation (treating patients away from society), avoidance, and hostile behavior (physical maltreatment or threats of harm) (4).

Stigma stands as one of the most significant contributors for diminishing the quality of life of mental patients and their families and as a barrier for the development of mental health care programs (5, 6).

The World Health Organization (WHO) has already pointed out some of the devastating consequences of stigma since it leads to social exclusion and isolation, hampers family relationships, limits social functioning, and favors human right abuse. These problems can be intensified by self-stigma that results from a process of internalization of public stereotypes, leading to decrement of self-esteem and self-efficacy and delays the search for psychiatry treatment and recovery (7, 8). It is known that people suffering from severe mental illness show a shorter life span and higher mortality rates compared to general population due to polypharmacy, physical illness, and suicide (9, 10). According to WHO one of the pivotal reasons explaining why people with mental problems have less access to health care is the stigma and discrimination associated with mental illness (8).

Alongside with general social stigma, the literature shows that stigmatizing attitudes toward patients with mental illness among mental health professionals and students exist in higher proportions than expected given the current knowledge on this topic (5, 11). Despite that, there is evidence that the literacy on mental health and the interaction with patients have positive effects on reducing stigma (11). This can be seen through the improvement on stigmatization scores as the students get more contact with mental health patients (12, 13).

There are many gaps in the research about stigma toward persons suffering from psychiatry disorders, mainly those intended to understand how it develops during medical education. The aim of the present study was to characterize and compare the presence of stigmatizing attitudes toward mental illness among medical students, psychiatrists, and non-psychiatry doctors in order to find if there are differences in attitude among different specialty and formation/working status.

## Methods

This is a non-interventional, observational, cross-sectional, and analytic study. The population assessed comprised the students of all classes of the Medical Degree of the University of Minho and medical doctors from psychiatry, internal medicine, and surgery working in public hospitals in Braga's region (Hospital de Braga, Hospital Senhora da Oliveira and Hospital de Fafe). All the participants signed a written informed consent, and the study protocol was approved by the ethics committee of School of Medicine.

Printed copies of a sociodemographic questionnaire and the Portuguese version of Attribution Questionnaire (AQ-27) (14) were given to the participants, and the answers were collected in ballots in order to ensure confidentiality.

The sociodemographic questionnaire included questions on age, gender, professional group, and information on previous personal and familiar experience of mental health disorders.

AQ-27 is a validated instrument designed to measure stigmatizing attitudes and reactions regarding nine dimensions: responsibility (patients with mental illness can control their condition and are responsible for it), pity (mental illness is beyond the control of the patients and they deserve other's sympathy), anger (patients with mental illness are blamed for their conditions and cause irritation and rage), dangerousness (people with mental illness are unpredictable and can be harmful for themselves and others), fear (patients with mental illness should be feared because they are dangerous), help (willingness to provide assistance people with mental illness), coercion (mandatory management of patients with mental illness), segregation (people with mental illness should be isolated from the community), and avoidance (effort to stay away from patients with mental illness). The items regarding responsibility, dangerousness, fear, anger, coercion, segregation, and avoidance can be associated with discriminatory behaviors in contrast with help and pity. This questionnaire contains a vignette of a patient with mental illness (in this case was a person suffering from schizophrenia) followed by 27 sentences that should be scored on a Likert scale ranging from 1 point (“no or nothing”) to 9 points (“very much or completely”). Higher factor scores represent greater endorsement of the corresponding attitude or belief.

The statistical analysis was performed using the *Statistical Package for the Social Sciences (SPSS) 19.0®* for *Windows®*. The AQ-27 dimensions were statistically compared between professional groups. The normality assumption was assessed using the Shapiro-Wilk test. If this assumption was met, one-way analysis of variance (ANOVA) was conducted; otherwise, the groups were compared with the non-parametric Kruskal-Wallis test. The differences between groups were determined using a *post hoc* Tukey test (*p* value was considered significant when <0.05). The contribution of individual variables on AQ-27 scores was assessed with linear regression modeling. For this, purpose demographic variables (age and gender), information on previous personal and familiar experience of mental health disorders and variables related to the professional group were set as independent variables. To account for the categorical nature of the professional group, two dummy variables were created: Student (1 if the participant is a student; 0 otherwise) and Psychiatrist (1 if the participant is a psychiatrist; 0 otherwise). This approach enables the use of categorical variables in the different regression models. Statistical significance was defined at the *p* < 0.05 level.

## Results

The sample included a total of 353 participants of which 203 (57.5%) were students, 121 (34.3%) were non-psychiatry doctors, and 29 (8.2%) were psychiatry specialists. The majority of the responders were female (65.2%, n = 230) and the age ranged from 17 to 73 (mean = 29.81; standard deviation (SD) = 12.42).

Global results obtained for each item evaluated on AQ-27 are shown in **Table 1**. Overall, coercion and avoidance were the dimensions that got the highest scores. Responsibility was the item with the lowest score (**Table 1**).

**Table 1:** Stereotypes means obtained in the AQ-27 in our sample, mean (SE).

	Psychiatrists	Non-Psychiatrists	Students
Gender (F/M)	17/11	58/59	155/48
Age	41.52 (2.49)	40.89 (1.04)	21.64 (0.21)
AQ-27 Responsibility	6.48 (0.64)	8.6 (0.38)	8.16 (0.27)
AQ-27 Fear	8.43 (0.65)	14.15 (0.59)	16.14 (0.36)
AQ-27 Help	24.41 (0.42)	19.04 (0.44)	22.07 (0.26)
AQ-27 Pity	16.68 (0.90)	16.25 (0.51)	18.56 (0.32)
AQ-27 Coercion	19.14 (0.50)	18.55 (0.34)	18.66 (0.24)
AQ-27 Segregation	10.55 (0.84)	16.18 (0.52)	16.81 (0.34)
AQ-27 Anger	12.71 (0.72)	14.95 (0.42)	13.79 (0.34)
AQ-27 Avoidance	6.17 (0.52)	10.61 (0.44)	10.75 (0.30)
AQ-27 Danger	13.38 (1.11)	19.58 (0.44)	14.74 (0.38)

**Figure 1:**
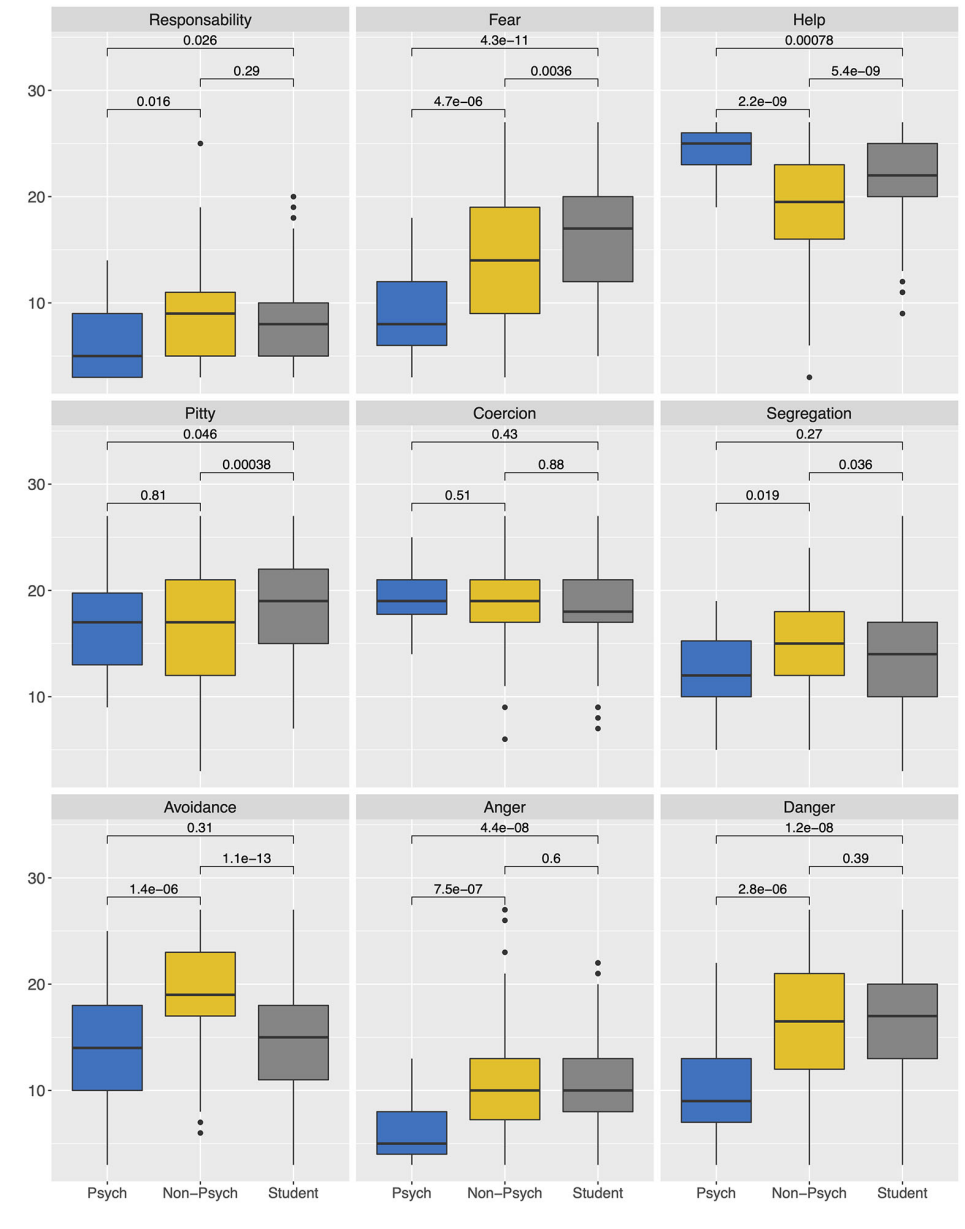
Comparison of AQ-27 score means for each stereotype according to the professional group.

The significance of the Shapiro-Wilk tests demonstrated that for most AQ-27 dimensions, there were statistically significant deviations from the normal distribution at least in one of the groups. Thus, the scores on these dimensions were compared using the Kruskal-Wallis test. The between-group differences are graphically represented in **Figure 1**. Psychiatrists displayed lower levels of stigmatizing attitudes in all categories analyzed, except “pity.” Students, on other hand, showed significantly lower stigmatizing attitudes in help, pity, and avoidance when compared with non-psychiatrist doctors. No differences were found between groups among coercion and segregation. All *p* values were corrected for multiple comparisons with Tukey test.

The regression analyses revealed statistically significant main effects for fear (F (6,337) = 8.523, *p* < 0.001), help (F (_6,339_) = 5.042, *p* < 0.001), pity (F _(6,339)_ = 5.121, *p* < 0.001), avoidance (F _(6,341)_ = 7.057, *p* < 0.001), anger (F _(6,341)_ = 3.909, *p* = 0.001) and dangerousness (F _(6,341)_ = 5.286, *p* < 0.000). When it comes to the contribution of the variables studied on the scores, we verified that the professional group was the one that showed more significant statistical differences in several stigmatizing attitudes like fear, help, avoidance, anger, and danger. Comparing to the remaining professional categories, being a psychiatrist is relevant to express significantly lower stigmatizing attitudes in fear, anger, and danger dimensions. When it comes to help and avoidance dimensions, both psychiatrists and students expressed fewer stigmatizing views than non-psychiatry doctors.

**Figure 2:**
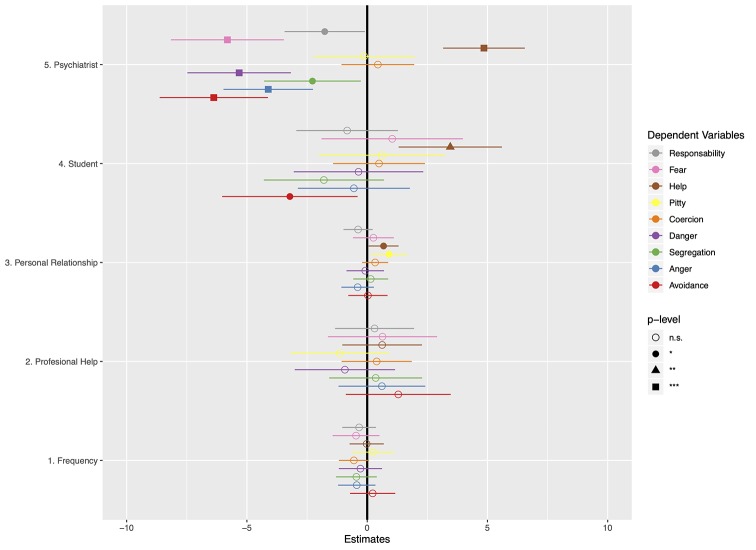
Graphical representation of the influence of each predictor on each AQ-27 score (*p* level: * < 0.05; ** < 0.01; *** < 0.001).

Age was an independent predictor of “pity,” with older participants revealing higher stigmatizing attitudes. The presence of a relative with mental illness also influenced significantly the scores on pity and help items by promoting fewer stigmatizing attitudes (**Table 2**; **Figure 2**).

**Table 2:** Regression Models: contribution of different variables on each AQ-27 score.

	Dependent Variable								
	Responsability	Fear	Help	Pity	Coercion	Segregation	Avoidance	Anger	Dangerousness
**Predictor**									
Constant	B = 9.081 (SE = 1.468), t = 6.187, *p* < 0.001	B = 16.792 (SE = 2.07), t = 8.113, *p* < 0.001	B = 19.267 (SE = 1.493), t = 12.903, *p* < 0.001	B = 19.705 (SE = 1.822), t = 10.816, *p* < 0.001	B = 17.838 (SE = 1.338), t = 13.327, *p* < 0.001	B = 16.248 (SE = 1.758), t = 9.243, *p* < 0.001	B = 17.553 (SE = 1.964), t = 8.938, *p* < 0.001	B = 11.924 (SE = 1.624), t = 7.344, *p* < 0.001	B = 17.562 (SE = 1.878), t = 9.35, *p* < 0.001
Psychiatrist	B = −1.765 (SE = 0.849), t = −2.078, *p* = 0.038	B=−5.811 (SE = 1.194), t=−4.867, *p* < 0.001	B = 4.854 (SE = 0.863), t = 5.622, *p* < 0.001	B = −0.128 (SE = 1.068), t = −0.12, *p* = 0.905	B = 0.443 (SE = 0.769), t = 0.577, *p* = 0.565	B = −2.275 (SE = 1.022), t = −2.227, *p* = 0.027	B = −6.378 (SE = 1.145), t=−5.573, *p* < 0.001	B = −4.114 (SE=0.946), t = −4.347, *p* < 0.001	B = −5.323 (SE = 1.095), t = −4.863, *p* < 0.001
Student	B = −0.833 (SE = 1.074), t=−0.775, *p* = 0.439	B = 1.039 (SE = 1.495), t = 0.695, *p* = 0.488	B=3.454 (SE=1.093), t=3.161, *p* = 0.002	B=0.603 (SE=1.335), t=0.452, *p* = 0.652	B = 0.492 (SE = 0.971), t = 0.506, *p* = 0.613	B = −1.801 (SE = 1.27), t = −1.417, *p* = 0.157	B = −3.215 (SE = 1.432), t = −2.245, *p* = 0.025	B = −0.553 (SE = 1.184), t = −0.467, *p* = 0.641	B = −0.361 (SE = 1.37), t = −0.263, *p* = 0.792
Age	B = −0.004 (SE = 0.028), t = −0.141, *p* = 0.888	B = −0.078 (SE = 0.04), t = −1.953, *p* = 0.052	B=−0.03 (SE=0.028), t=−1.066, *p* = 0.287	B=−0.081 (SE=0.035), t=−2.308, *p* = 0.022	B = 0.015 (SE = 0.025), t = 0.591, *p* = 0.555	B = −0.03 (SE = 0.034), t = −0.887, *p* = 0.376	B = 0.019 (SE = 0.037), t = 0.493, *p* = 0.622	B = −0.029 (SE = 0.031), t = −0.92, *p* = 0.358	B = −0.018 (SE = 0.036), t =−0.5, *p* = 0.617
Gender	B = −0.051 (SE = 0.206), t = −0.247, *p* = 0.805	B = 0.172 (SE = 0.284), t = 0.605, *p* = 0.545	B=0.027 (SE=0.231), t=0.119, *p* = 0.906	B=−0.193 (SE=0.256), t=−0.754, *p* = 0.451	B=−0.054 (SE=0.183), t=−0.296, *p* = 0.767	B=−0.158 (SE=0.241), t=−0.657, *p* = 0.512	B = 0.073 (SE = 0.277), t = 0.265, *p* = 0.791	B = 0.06 (SE = 0.229), t = 0.262, *p* = 0.793	B = 0.371 (SE = 0.265), t = 1.402, *p* = 0.162
Personal relation	B = −0.379 (SE = 0.309), t = −1.23, *p* = 0.22	B = 0.258 (SE = 0.432), t=0.598, *p* = 0.55	B = 0.681 (SE = 0.317), t = 2.146, *p* = 0.033	B = 0.91 (SE = 0.387), t = 2.352, *p* = 0.019	B = 0.33 (SE = 0.277), t = 1.19, *p* = 0.235	B = 0.147 (SE = 0.369), t = 0.397, *p* = 0.691	B = 0.03 (SE = 0.415), t = 0.073, *p* = 0.942	B = −0.397 (SE = 0.343), t = −1.158, *p* = 0.248	B = −0.08 (SE = 0.397), t = −0.202, *p* = 0.84
Frequency	B = −0.335 (SE = 0.357), t=−0.939, *p* = 0.348	B=−0.464 (SE=0.495), t=−0.938, *p* = 0.349	B=−0.021 (SE=0.361), t=−0.059, *p* = 0.953	B=0.237 (SE=0.443), t=0.535, *p* = 0.593	B = −0.553 (SE = 0.318), t = −1.74, *p* = 0.083	B=−0.451 (SE = 0.432), t = −1.044, *p* = 0.297	B = 0.227 (SE = 0.479), t = 0.474, *p* = 0.636	B = −0.433 (SE = 0.396), t = −1.095, *p* = 0.274	B = −0.28 (SE = 0.458), t = −0.613, *p* = 0.54
Profissional help	B = 0.306 (SE = 0.836), t = 0.366, *p* = 0.714	B = 0.636 (SE=1.155), t = 0.551, *p* = 0.582	B = 0.622 (SE = 0.842), t = 0.739, *p* = 0.461	B = −1.144 (SE = 1.041), t = −1.099, *p* = 0.272	B = 0.395 (SE = 0.742), t = 0.532, *p* = 0.595	B = 0.354 (SE = 0.982), t = 0.36, *p* = 0.719	B=1.292 (SE=1.109), t=1.165, *p* = 0.245	B = 0.612 (SE = 0.917), t = 0.667, *p* = 0.505	B = −0.927 (SE = 1.061), t = −0.874, *p* = 0.383
**Model summary**									
	F (7,337) = 1.401, *p* = 0.204; R2 = 0.028; R2adj = 0.008	F (7,336) = 8.25, *p* = 0.000; R2 = 0.147; R2adj = 0.129	F (7,338) = 9.72, *p* = 0.000; R2 = 0.168; R2adj = 0.15	F (7,338) = 4.388, *p* = 0.000; R2 = 0.083; R2adj = 0.064	F (7,337) = 0.835, *p* = 0.559; R2 = 0.017; R2adj = −0.003	F (7,334) = 1.294, *p* = 0.253; R2 = 0.026; R2adj = 0.006	F (7,340) = 10.274, *p* = 0.000; R2 = 0.175; R2adj = 0.158	F (7,340) = 4.737, *p* = 0.000; R2 = 0.089; R2adj = 0.07	F (7,340) = 6.1, *p* = 0.000; R2 = 0.112; R2adj = 0.093

## Discussion

The present study aimed to characterize stigmatization attitudes among medical students, psychiatrists, and non-psychiatry doctors, and it showed that psychiatrists hold the lowest scores on stigmatization levels (except for coercion), followed by students and doctors of other specialties.

Our observation that psychiatrists have the least negative attitudes toward patients with mental illness comes in line with others studies that show the same conclusion (15). Particularly, the difference with other doctors who have higher levels of stigmatization may be explained by the *contact hypothesis*. Psychiatrists as a professional group have more personal contact with mental illness and that has been proven to significantly reduce stigma and enhance positive approach to it (16, 17). In the same vein, physicians who have a relative with mental illness also expressed fewer stigmatizing attitudes with significantly higher scores on pity and help. Together, these results are in line with convincing evidence that increased contact with people suffering from mental illness is associated with lower stigma (15–17).

Another factor that can help to understand this difference is the *physicians bias* that states that the attitudes held by a health provider may be conditioned by training and/or past experiences with patients with mental illness. We hypothesize that doctors from other specialties may have contact with more complicated patients that have to be seen in emergency room setting with self-inflicted lesions or disruptive conduct in virtue of severe psychiatry illness. Our study showed a statistically significant higher score on avoidance that may be related to the aforementioned factors.

Students' scores are placed in an intermediate level between psychiatrists and non-psychiatrists. As previously shown by several studies, older people are more prone to engage in stigmatizing attitudes toward mental patients (18, 19). Our study showed similar results once age appeared as an independent factor in the regression: being younger, students will present a more positive attitude. Moreover, psychiatrists included in this study coach students from the medical school. Interestingly, there are studies that show that when professors exhibit less stigmatizing attitudes, as shown in our study, student's negative attitudes will tend to improve toward both psychiatry and patients with mental illness (16). Plus, some of the students in our sample had already attended psychiatry rotation which makes them more prone to change the way they view psychiatric patients. Studies comparing pre- and post-clinical students demonstrate that as the level of education in psychiatry rises, the level of stigmatization decreases (17).

Even though our results are in accordance with the literature, it is relevant to point out that the group we studied relied on a convenient sample that included students and doctors of a particular geographic area. The same applies to students from this particular medical school, which includes a specific program of psychiatry training that offers early contact with psychiatric patients and simulated clinical consultations of different psychiatric syndromes (20). Furthermore, being a cross-sectional study, it does not allow to follow the changes of student's attitudes over time, considering that students from different degrees of the medical course were included together as a group. Other limitations of the study include the use of preliminary version of the AQ-27 in Portuguese, the limited variability of the samples, and the unequal sample size for each group. Additionally, it might be interesting in future research to compare medical students/professionals' stigma with other health professionals and the general population.

## Conclusions

In brief, this study shows that stigmatization still exists inside medical community. Psychiatrists presented lower levels of stigma compared with non-psychiatry physicians and medical students. We found that stigma is related with is related with age, lower professional contact with persons suffering from psychiatric disorders and the presence of a relative with mental health disorders. Thus, interventions regarding this matter are crucial to bring insight about the negative impact of stigmatization against patients with mental illness. Measures like changing the curriculum of medical schools in order to lecture on this topic to the students and promote contact with patients with psychiatric disorders could prove beneficial to break stereotypes and to reduce the negative consequences of stigma. Finally, psychiatrists should promote educational interventions among other medical specialties in order to reduce stigma against psychiatry itself.

## Data Availability Statement

The datasets generated for this study are available on request to the corresponding author.

## Ethics Statement

The study was conducted according to Declaration of Helsinki and obtained ethical approval from the Board of School of Medicine of University of Minho. Written informed consent was obtained from all the participants.

## Author Contributions

PM, FP, JC, and NS designed the study. AO, DM, and PSM collected the data. JF, PSM, and JC analyzed the data. All authors contributed to the writing of the manuscript. All authors reviewed and edited the final version of the manuscript. All authors read and approved the final manuscript.

## Funding

This work was partially funded by the FEDER funds, through the Competitiveness Factors Operational Programme (COMPETE), and by national funds, through the Foundation for Science and Technology (FCT), under the scope of the project UID/Multi/50026/2019. This manuscript has been developed under the scope of the project NORTE-01-0145-FEDER-000013, supported by the Northern Portugal Regional Operational Programme (NORTE 2020), under the Portugal 2020 Partnership Agreement, through the European Regional Development Fund (FEDER).

## Conflict of Interest

The authors declare that the research was conducted in the absence of any commercial or financial relationships that could be construed as a potential conflict of interest.

## Abbreviations

AQ-27, Portuguese version of Attribution Questionnaire; WHO, World Health Organization.

## References

[1] Lauber C Stigma and discrimination against people with mental illness: a critical appraisal. Epidemiol Psychiatr Sci (2008) 17:10–3. 10.1017/S1121189X0000261X 18444451

[2] Ostman M Kjellin L Stigma by association: psychological factors in relatives of people with mental illness. Br J Psychiatry: J Ment Sci (2002) 181:494–8. 10.1192/bjp.181.6.494 12456519

[3] Corrigan PW Mental Health Stigma as Social Attribution: Implications for Research Methods and Attitude Change. Clin Psychol Sci Pract (2000) 7(1):48–67. 10.1093/clipsy.7.1.48

[4] Corrigan P Markowitz FE Watson A Rowan D Kubiak MA An attribution model of public discrimination towards persons with mental illness. J Health Soc Behav (2003) 44(2):162–79. 10.2307/1519806 12866388

[5] Sartorius N Stigma: what can psychiatrists do about it? Lancet (1998) 352(9133):1058–9. 10.1016/S0140-6736(98)08008-8 9759771

[6] Henderson C Evans-Lacko S Thornicroft G Mental Illness Stigma, Help Seeking, and Public Health Programs. Am J Public Health (2013) 103(5):777–80. 10.2105/AJPH.2012.301056 PMC3698814 23488489

[7] Watson AC Corrigan P Larson JE Sells M Self-stigma in people with mental illness. Schizophr Bull (2007) 33(6):1312–8. 10.1093/schbul/sbl076 PMC2779887 17255118

[8] Sartorius N Stigma and mental health. Lancet (2007) 370(9590):810–1. 10.1016/S0140-6736(07)61245-8 17804064

[9] Hayes JF Miles J Walters K King M Osborn DP A systematic review and meta-analysis of premature mortality in bipolar affective disorder. Acta Psychiatrica Scand (2015) 131(6):417–25. 10.1111/acps.12408 PMC4939858 25735195

[10] Hayes JF Marston L Walters K King MB Osborn DPJ Mortality gap for people with bipolar disorder and schizophrenia: UK-based cohort study 2000-2014. Br J Psychiatry: J Ment Sci (2017) 211(3):175–81. 10.1192/bjp.bp.117.202606 PMC5579328 28684403

[11] Spagnolo AB Murphy AA Librera LA Reducing stigma by meeting and learning from people with mental illness. Psychiatr Rehabil J (2008) 31(3):186–93. 10.2975/31.3.2008.186.193 18194945

[12] Telles-Correia D Marques JG Gramaça J Sampaio D Stigma and Attitudes towards Psychiatric Patients in Portuguese Medical Students. Acta Médica Portuguesa (2015) 28(6):715–9. 10.20344/amp.6231 26849755

[13] Marques A Barbosa T Queirós C Stigma in mental health: perceptions of students who will be future health professionals. Eur Psychiatry (2011) 26(Supplement 1):1439. 10.1016/S0924-9338(11)73144-3

[14] Sousa S Marques A Rosario C Queiros C Stigmatizing attitudes in relatives of people with schizophrenia: a study using the Attribution Questionnaire AQ-27. Trends Psychiatry Psychother (2012) 34(4):186–97. 10.1590/S2237-60892012000400004 25923067

[15] Corrigan PW Powell KJ Michaels PJ Brief battery for measurement of stigmatizing versus affirming attitudes about mental illness. Psychiatry Res (2014) 215(2):466–70. 10.1016/j.psychres.2013.12.006 24388505

[16] Griffiths KM Carron-Arthur B Parsons A Reid R Effectiveness of programs for reducing the stigma associated with mental disorders. A meta-analysis of randomized controlled trials. World Psychiatry (2014) 13(2):161–75. 10.1002/wps.20129 PMC4102289 24890069

[17] Eksteen HC Becker PJ Lippi G Stigmatization towards the mentally ill: Perceptions of psychiatrists, pre-clinical and post-clinical rotation medical students. Int J Soc Psychiatry (2017) 63(8):782–91. 10.1177/0020764017735865 29067838

[18] Hansson L Stjernsward S Svensson B Changes in attitudes, intended behaviour, and mental health literacy in the Swedish population 2009-2014: an evaluation of a national antistigma programme. Acta Psychiatrica Scand (2016) 134 Suppl 446:71–9. 10.1111/acps.12609 27426648

[19] Winkler P Csemy L Janouskova M Mlada K Bankovska Motlova L Evans-Lacko S Reported and intended behaviour towards those with mental health problems in the Czech Republic and England. Eur Psychiatry (2015) 30(6):801–6. 10.1016/j.eurpsy.2015.05.003 26113172

[20] Pereira VH Morgado P Goncalves M Costa L Sousa N Cerqueira JJ An Objective Structured Clinical Exam to Assess Semiology Skills of Medical Students. Acta Med Port (2016) 29:819–25. 10.20344/amp.8407 28425885

